# One size does not fit all: working towards increasing participation of minority groups in cancer screening programmes

**DOI:** 10.1186/s12916-023-02756-3

**Published:** 2023-03-16

**Authors:** BMC Medicine

**Affiliations:** Springer Nature, London, UK

Cancer is the second leading cause of death worldwide, which according to WHO claimed nearly 10 million lives in 2020. Early diagnosis of cancer increases the chance of successful treatment and patient survival. The introduction of free cancer screening programmes has been a monumental step towards early diagnosis and potential prevention of different cancers. These strategies have also become a beacon of hope for lowering the amount of people on this perilous path and therefore reducing the global cancer burden. The UK currently offers national screening programmes for breast, cervical and bowel cancer to different age groups. In 2019, the review report by NHS England revealed that these services save almost 9000 lives each year through prevention and early diagnosis. However, despite the vast investments from the government to roll out continuously improving tests to the public, some ethnic demographics are reluctant to participate in screenings. For example, a 5-year analysis of the English Colorectal Cancer Screening Programme found that uptake was nearly 14% lower in the most deprived areas and almost 16% lower in the most ethnically diverse areas compared to the least deprived and least ethnically diverse areas. Participation in the cervical screening programme in Denmark was also found to be 13% lower in migrant women than in Danish-born women. This, unfortunately, has the potential to undermine the effectiveness of screening programmes and drive the issue of inequalities in healthcare. So, why is there less uptake by ethnic minorities in cancer screening programmes? More importantly, how can different communities be navigated towards acknowledging the necessity of these simple yet potentially life-saving tests, so their interests to partake in them can be matched to the rest of the public, thus eventually closing this lingering gap?

Several factors have been identified as the driving forces behind the lower numbers of ethnic minorities and socioeconomically deprived populations participating in different screening tests which contribute to the existing gap. Some common examples include lack of knowledge, fear or shame/stigma, lack of time, general mistrust in the healthcare system, anxiety surrounding test results, influence of family and friends, religious beliefs, low perceived risk or absence of symptoms, discomfort or pain and health insurance. All these points highlight the importance of raising awareness about screening tests and the outstanding misconceptions within different communities. However, they also imply that individuals and their decision to uptake screening tests can be influenced by personal or cultural beliefs. Therefore, community leaders with connections to the beliefs and cultures of such groups can have a prominent role in guiding them. Practical barriers such as lack of time largely arise from work and childcare commitments which push the screening tests further down the priority lists for such busy patients, particularly if not much flexibility exists around healthcare centre opening times and pre-allocated appointments. What’s interesting is that the aforementioned findings have come from a breadth of countries, and in most cases, there are shared concerns amongst the patients involved in different studies. One example is the attitude and shared concerns of ethnic minority women towards cervical cancer screening in the UK, Denmark, Norway and Australia which act as a barrier to their uptake of the tests. This redirects the focus from the issue being country of residence related towards something a little deeper rooted.

Some steps have been taken to explore potential interventions to address the gaps in cervical cancer screening, with most focusing on increasing participation of ethnic minority women in cervical cancer screening programmes. Education with cultural adaptation appears to be at the forefront of interventions employed to encourage partaking in screening programmes. Some popular methods used have been counselling delivered through group education, one-to-one sessions and small media. The SWIM study found that ethnic minority women were interested in a tailored intervention involving an educational strategy with a positive tone and clear message conveyed through group teaching sessions held by a health-care professional in addition to a communication strategy with information in their native language. Other recommendations by the patients were illustrations as well as strategies to increase awareness about cervical cancer screening and involve the women in actively spreading the word about the screening offer in their network. Although some studies have found improvements in cancer knowledge, attitudes, intention and perceptions and health beliefs, there is still inadequate data on their impact on actual patient participation in cancer screening programmes.

This World Cancer Day, we must acknowledge the efforts made by healthcare professionals to reduce cancer burden and appreciate the ground-breaking contribution that public cancer screening programmes have made to the survival of patients. Countries like the UK deserve credit for practising the roll-out of free cancer screening tests in their healthcare system. Whilst the issue of lower participation from minority groups in screening programmes is worrying, it is not unresolvable. Despite all the research done in the area of cancer screening uptake by different populations in the past decade, the continuous existence of the inequality gap raises questions about whether sufficient attempts are being made by governments, health providers and public to implement the findings and potential solutions highlighted in different studies into practice to reduce this gap. More data needs to be acquired on the participation of different communities in cancer screening programmes in different countries to understand better where the inequality gap stands. Frequent national-level reviews on the effectiveness of cancer screening programmes and their uptake by different minority and socioeconomically deprived groups are needed by health systems to monitor how the gap is changing over time. This may also enable countries to determine the impact of the studies on barriers to cancer screening uptake and any relevant interventions on their populations.

Gaps in minority cancer screening participation can occur in any country regardless of economic status. Whilst it is undeniable that the roll-out of public cancer screening tests requires extensive investment, their importance is essential. Changing the level of uptake of these screening resources by groups which do not typically utilise them could be a game-changer. Such change could become one of the biggest steps towards potentially reversing the growing trend of global cancer burden and changing history.

